# Inequities in quality and safety outcomes for hospitalized children with intellectual disability

**DOI:** 10.1111/dmcn.15066

**Published:** 2021-09-25

**Authors:** Laurel Mimmo, Reema Harrison, Joanne Travaglia, Nan Hu, Susan Woolfenden

**Affiliations:** ^1^ School of Population Health Faculty of Medicine University of New South Wales Sydney New South Wales Australia; ^2^ Clinical Governance Unit The Sydney Children’s Hospitals Network Sydney New South Wales Australia; ^3^ Health Management Programs Faculty of Medicine School of Population Health University of New South Wales Sydney New South Wales Australia; ^4^ Faculty of Health Centre for Health Services Management University of Technology Sydney Sydney New South Wales Australia; ^5^ Faculty of Medicine School of Women’s and Children’s Health University of New South Wales Sydney New South Wales Australia; ^6^ Community Child Health Sydney Children’s Hospital, Randwick Sydney New South Wales Australia

## Abstract

**Aim:**

To investigate if there are inequities in quality and safety outcomes for children with intellectual disability admitted to two tertiary paediatric hospitals.

**Method:**

A cross‐sectional study of 1367 admissions for 1018 randomly selected patients admitted for more than 23 hours to one of two tertiary children’s hospitals in Sydney, Australia (1st January–31st December 2017). Electronic medical records were manually interrogated to identify children with intellectual disability (including developmental delay). Data extracted included patient demographics, length of stay, number of admissions, and reported clinical incidents.

**Results:**

In total, 12.3% (*n*=125) of children admitted during the study period had intellectual disability, which represented 13.9% (*n*=190) of admissions. Sex and age at admission in children with and without intellectual disability were similar: 83 (43.7%) vs 507 (43.1%) females and 107 (56.3%) vs 670 (56.9%) males, *p*=0.875; median age 3 years (0–18y) vs 4 years (0–18y), *p*=0.122. Children with intellectual disability had significantly greater median length of stay (100.5h vs 79h, *p*<0.001) and cost of admission (A$11 596.38 vs A$8497.96) than their peers (*p*=0.001). Children with intellectual disability had more admissions with at least one incident compared to children without intellectual disability (14.7% vs 9.7%); this was not statistically significant (*p*=0.06).

**Interpretation:**

Children with intellectual disability experience inequitable quality and safety outcomes in hospital. Engaging children and families in clinical incident reporting may enhance understanding of safety risks for children with intellectual disability in hospital.

## Abstract 2

This original article is commented by Tenenbaum on page 279 of this issue.

**What this paper adds**

- Children with intellectual disability have high health care utilization rates yet were not routinely identified when accessing hospitals in this study.
- When admitted to hospital, children with intellectual disability experienced longer median length of stay and higher cost of admission than their peers.
- Children with intellectual disability had more admissions with at least one incident compared to children without intellectual disability.

Approximately 1% of the global population has intellectual disability with higher rates in children and adolescents.^1^ In Australia, 4.5% of children aged younger than 15 years have intellectual disability.^2^ Intellectual disability is diagnosed in individuals younger than 18 years who demonstrate permanent impairments to learning, thinking and reasoning, and social functioning.^3^ Children with intellectual disability have high rates of chronic comorbid health conditions that require specialist care such as epilepsy, cerebral palsy, and mental health disorders.^4^, ^5^ Because of their complex health care needs these children often have higher rates of health care utilization than the general population including hospital admissions^6^, ^7^ and may need to be hospitalized for longer, although the data on this is limited.

Optimizing the quality and safety of health care is the cornerstone of high performing health systems globally; poor care quality and safety result in avoidable harm and preventable deaths in patients, and increased costs for the health system.^8^ There is evidence that children with complex care needs have higher rates of poor quality and safety outcomes during their hospital stay, particularly for those from socioeconomically disadvantaged and culturally and linguistically diverse backgrounds.^9^, ^10^, ^11^

While there is qualitative data that has found that children with intellectual disability and their parents/carers report poor quality of care experiences in hospital,^12^, ^13^ there is limited larger scale evidence from quantitative studies regarding inequities in health care quality and safety for children with intellectual disability at system or service level.^14^, ^15^, ^16^ For example, our earlier work identified that the length of stay for children coded with intellectual disability, according to the International Classification of Diseases, 10th Revision (ICD‐10)^17^ Australian modification, was 23 hours longer than children not coded with intellectual disability.^18^ Given that longer length of stay is associated with health care harms in tertiary paediatric settings,^9^ it is possible that longer length of stay amongst children with intellectual disability might expose them to more opportunites for harm in their care.

Conversely, longer length of stay amongst this population may be masking latent safety concerns. An English study exploring adverse events in people with intellectual disability in hospital found health care staff did not easily recognize patient safety risks in this population.^19^ To date, there is lack of evidence regarding both the extent to which children with intellectual disability are reliably identified when they are admitted to hospital and also the safety of their care. Central to understanding and effectively addressing quality and safety issues in children with intellectual disability is the reliable measurement and reporting of quality and safety outcomes for this population.

The present study aimed to address this critical gap by investigating if there are inequities in quality and safety outcomes for children aged 0 to 18 years with intellectual disability admitted to two tertiary paediatric hospitals. The study objectives were to: (1) quantify the prevalence of the paediatric intellectual disability population admitted to two tertiary children’s hospitals; (2) compare quality and safety outcomes for children with intellectual disability compared to those without intellectual disability and any associated risk factors such as number of admissions and geographic location.

## METHOD

### Ethics

Research ethics approval for this study was granted by The Sydney Children’s Hospitals Network human research ethics committee (reference number: 2019/ETH00367) and the Aboriginal Health & Medical Research Council of New South Wales (reference number: 1541/19).

### Participants and setting

This was a retrospective chart review of electronic medical records for 1021 randomly selected patients from a total of 21 337 patients aged 0 to 18 years admitted for longer than 23 hours to two tertiary children’s hospitals in Sydney, Australia in 2017. A random sample was necessary as there is currently no flag or alert within the medical record to indicate a patient has intellectual disability; a manual search of each patient record was required. To maintain focus on overnight admissions to inpatient ward areas we excluded admissions of less than 23 hours, admissions where the child remained in the emergency department for observation, and admissions from other hospitals for surgical procedures where the child returned to the referring hospital within 24 hours of the procedure. These different contexts for care delivery present different quality and safety experiences and warrant separate study.^8^

**Figure 1:**
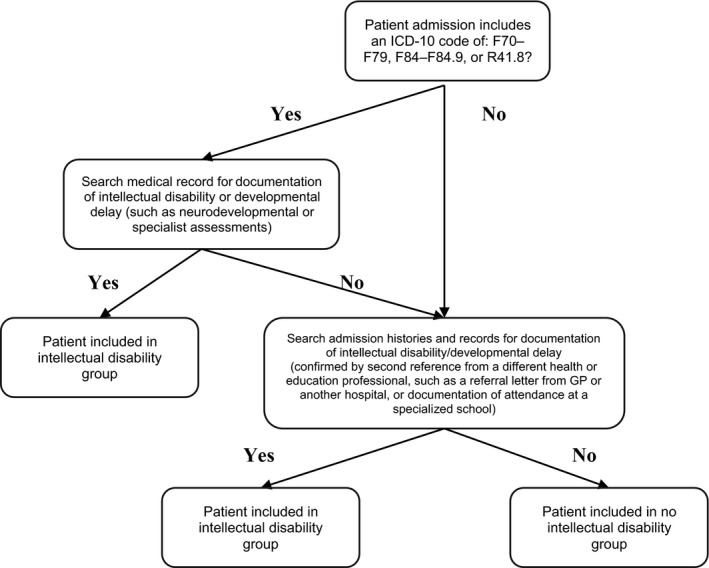
Flowchart for identifying children with intellectual disability in the medical record.

The random selection of patients was conducted by the health service medical record data analyst. The data analyst created a list of medical records for each patient admitted for greater than 23 hours during the study period and selected every 16th patient for inclusion in the sample (see Fig. 1). The admission details for each of the selected patients was then forwarded to LM (see Appendix S1, online supporting information). The prevalence rate of children aged 0 to 14 years with intellectual disability in the general population in Australia of 4.5%, based on 2018 survey data from the Australian Bureau of Statistics,^2^ was used to ensure that the sample size was sufficiently powered. Based on this information, the sample size of 1018 patients from 21 337 patients admitted for greater than 23 hours in 2017 has enabled us to detect a minimum 44% increase in this prevalence for the general population. That is, to detect at least a 6.5% prevalence of intellectual disability in the admitted population compared to the general population (4.5%), with 80% power and two‐sided significance level of 5%.

### Exposure group: children with intellectual disability

To identify if the patient had a documented intellectual disability each record was systematically searched by a single nurse reviewer (LM) to identify documentation of intellectual disability or global developmental delay. If such documentation was not available, the medical, nursing, and allied health admission histories were reviewed for documentation of intellectual disability, and confirmed by a second reference from a different health or education professional. In addition, where a child was documented as completing their final year of high school with a leaving certificate and/or there was no other mention of school, cognitive skill, or development, it was assumed the child did not have intellectual disability. This process is summarized in Figure 1 and Appendix S1. For preschool children aged younger than 6 years, a child was categorized in the developmental delay group only where there was documented evidence of significant developmental delays identified through validated developmental assessments such as Bayley, Griffiths, or Wechsler scales.

Patients were then categorized into one of three groups, based on clinical documentation from the medical record: no intellectual disability or developmental delay, intellectual disability, and developmental delay. For analysis and reporting, the intellectual disability group includes those with developmental delay. We included the developmental delay group to account for children aged less than 6 years (Appendix S1); in the preschool years diagnosing intellectual impairment is often deferred to school age unless a child has been diagnosed with a specific disorder or condition known to be associated with intellectual disability.^20^ Children with autism spectrum disorder who did not have any documented evidence of intellectual disability were included in the no intellectual disability group, as these are distinct populations.^21^

### Outcome measures: quality and safety

#### Length of stay and cost

A routinely used quality indicator for inpatient care is length of stay;^22^ prolonged length of stay is also associated with adverse events in paediatric settings.^9^, ^23^ We reported on median cost of admission as an additional indicator of quality and safety; adverse events from health care contribute significant cost burden to health systems worldwide.^24^

#### Clinical incident

Patient safety processes are used to report, categorize, and monitor clinical incidents according to local organizational policy. In the New South Wales health system a clinical incident that occurs during the provision of health care, for example medication errors, patient falls, pressure injuries, miscommunication, wrong site surgery, etc, is defined as ‘Any unplanned event resulting in, or with the potential for, injury, damage or other loss. This includes a near miss’.^25^ We reported on rates of reported clinical incidents. In New South Wales Health organizations, clinical incident type categories are predetermined in the Incident Investigation and Management System database. A reported clinical incident can have more than one incident type associated with the incident;^25^ for example, a medication administration error can be categorized as both medication and clinical management type. For the purpose of this analysis we report here the primary incident type associated with each incident. Patient aggression incidents not associated with care delivery were excluded from the analysis.

### Data collection

One nurse reviewer with expertise in paediatric health care quality (LM) searched each electronic record to determine if the patient had a documented intellectual disability using the process outlined above. Once data collection was complete, the developmental delay group was incorporated into the intellectual disability group for analysis and reporting. Where there was uncertainty about the presence of intellectual disability or developmental delay and/or documentation was unclear, the patient was discussed and their intellectual disability status clarified with a second medical reviewer (SW) who is a developmental paediatrician.

### Statistical analysis

SPSS version 26 (IBM Corp., Armonk, NY, USA), Stata version 15 (Statacorp, College Station, TX, USA), and SAS (Enterprise Guide) statistical software version 7.1 (SAS Institute Inc., Cary, NC, USA) programs were used for statistical analysis. The prevalence of intellectual disability was reported as a proportion with a 95% confidence interval (CI). Demographic variables were reported as means and standard deviations for continuous variables and proportions for categorical variables; age was reported as median with interquartile range (IQR). Length of stay and cost of admission was reported as median and IQR. χ^2^ test was used for categorical variables such as those shown in Table 1. A Mann–Whitney *U* test was used to test for difference in length of stay and cost of admission between children with and without intellectual disability. Logistic regression models were fitted to examine if children with intellectual disability were more likely to have more admissions involving a clinical incident compared to children without. Considering the potential clustering of admissions within the same patient, generalized estimating equations approach was used in the regression analysis using the empirical estimates for standard errors.

**Table 1:** Prevalence, demographics, admission patterns, length of stay, and cost of admission for random sample of 1018 patients admitted to The Sydney Children’s Hospitals Network for greater than 23 hours in 2017

Demographics	Number of admissions for 1018 patients	Admissions for children with intellectual disability or developmental delay	Admissions for children without intellectual disability or developmental delay	*p*
Prevalence (%; 95% CI)	1367 (100)	190 (13.89; 12.07–15.73)	1177 (86.10; 84.27–87.94)	
Sex				
Female	590 (43.16)	83 (43.68)	507 (43.08)	0.875
Male	777 (56.84)	107 (56.32)	670 (56.92)	
Median age (IQR, range), y		3 (1–8; 18)	4 (0–10; 18)	0.122
Country of birth				
Australia	1259 (92.1)	173 (91.1)	1086 (92.3)	0.564
Overseas	108 (7.9)	17 (8.9)	91 (7.7)	
English spoken at home				
Yes	1193 (87.3)	160 (84.2)	1033 (87.8)	0.172
No	174 (12.7)	30 (15.8)	144 (12.2)	
Location				
Metropolitan	1181 (86)	153 (81)	1028 (87)	0.012
Rural	115 (8)	26 (14)	89 (8)	
Interstate, ACT	35 (3)	7 (4)	28 (2)	
Interstate (excludes ACT)	8 (<1)	2 (1)	6 (<1)	
Overseas	28 (2)	2 (1)	26 (2)	
Number of admissions per patient				
1	845 (83.01)	93 (74.4)	752 (84.2)	0.018
2–3	132 (12.97)	23 (18.4)	109 (12.21)	
4 or more	41 (4.03)	9 (7.2)	32 (3.58)	

Data are *n* (%) unless otherwise stated. CI, confidence interval; IQR, interquartile range; ACT, Australian Capital Territory.

## RESULTS

### Prevalence of intellectual disability admitted to hospital

In 2017, 21 337 children were admitted to the two tertiary children’s hospitals in Sydney, Australia for more than 23 hours. From the 1021 randomly sampled children, three were excluded from the study as they were not admitted to acute ward areas. Of the remaining 1018 patients, 125 (12.3%, 95% CI: 10.3–14.3%) had documented evidence of intellectual disability; there was no documented evidence of intellectual disability in the remaining 893 (87.7%, 95% CI: 85.7–89.7%) children. The sample of 1018 children had 1367 admissions of greater than 23 hours during the study period. Of the 1367 admissions, 190 (13.9%, 95% CI: 12.1–15.7%) were for children with intellectual disability, indicating this cohort had a greater health utilization rate than their peers, who had 1177 (86.1%, 95% CI: 84.3–87.9%) admissions during the study period (*p*<0.001). The prevalence is depicted as a flowchart in Figure S1 (online supporting information).

### Number of admissions

For all children in the study sample (*n*=1018), 83% had one admission during the study period, 13% had two or three admissions, 4% had four or more admissions. Children with intellectual disability had a lower proportion of single admissions during the study period (74.4% vs 84.2%) than children without intellectual disability. Children with intellectual disability were significantly more likely to have two to three (18.4% vs 12.2%) or four or more (7.2% vs 3.5%) admissions during the study period (*p*=0.018; Table 1).

### Demographic data

Demographic data are presented in Table 1. There was no difference in sex between the two groups. Median age at admission was similar across the two groups (*p*=0.122). Children with intellectual disability had a median age of 3 years (IQR: 1–8; range: 0–18). Children without intellectual disability had a median age of 4 years (IQR: 0–10; range: 0–18).

The majority of children in the total sample came from a metropolitan location (86%), though this was less likely amongst children with intellectual disability (81%) than children without intellectual disability (87%). Admissions for children with intellectual disability were significantly more likely to be from rural locations than children without intellectual disability (14% vs 8%, *p*=0.012).

#### Quality and safety indicators

##### Length of stay

Children with intellectual disability had a median length of stay that was 21.5 hours longer than children without intellectual disability (100.5h vs 79h, *p*<0.001).

##### Cost of admission

Children with intellectual disability had a median cost of admission to hospital that was A$3098.42 more than the median cost for a child without intellectual disability (A$11 596.38 vs A$8497.96, *p*=0.001).

##### Reported clinical incidents

There were 211 incidents reported for 142 admissions of the total 1367 admissions during the study period or 10.4% (142/1367) of admissions. Children with intellectual disability had more admissions with at least one incident (14.7%, 28/190) compared to children without intellectual disability (9.7%, 114/1177), however this difference was not statistically significant (*p*=0.06).

##### Types of reported incidents

For all admissions, the top three reported primary incident types were medication/intravenous fluid (86/211, 41%), clinical management (48/211, 23%) and documentation (15/211, 7%). The top three primary incident types involving children with intellectual disability were the same, though there was a higher proportion of medication/intravenous fluid (21/44, 48%) and documentation (5/44, 11%) incidents, and a lower proportion of clinical management (7/44, 16%). For children without intellectual disability the top three primary incident types were medication/intravenous fluid (65/167, 39%), clinical management (41/167, 25%) and blood/blood products (15/167, 9%). Proportions for all reported primary incident types are presented in Table 2.

**Table 2:** Quality and safety indicators for random sample of 1018 patients admitted to The Sydney Children’s Hospitals Network of >23 hours in 2017

	All admissions (*n*=1367)	Children with intellectual disability or developmental delay (*n*=190)	Children without intellectual disability or developmental delay (*n*=1177)	*p*
Median length of stay (IQR, range), h		100.5 (55–231.5; 2375)	79 (50–148; 9160)	<0.001
Median cost of admission (IQR, range), $AUS		11 596.38 (5946.26–24 349.67; 388 963.81)	8497.96 (5441.66–16 566.81; 1 642 059.94)	0.001
Reported clinical incidents				
Admissions with no incident reported	1225 (89.6)	162 (85.3)	1 063 (90.3)	0.06
Admissions with at least one incident reported	142 (10.4)	28 (14.7)	114 (9.7)	0.06
Reported incidents by primary incident type				
Accident/occupational health and safety	2 (1)	0 (0)	2 (1)	
Aggression – aggressor	0 (0)	0 (0)	0 (0)	
Anaesthesia	0 (0)	0 (0)	0 (0)	
Behaviour/human performance	2 (1)	1 (2)	1 (<1)	
Blood/blood product	17 (6)	2 (5)	15 (9)	
Clinical management	48 (23)	7 (16)	41 (25)	
Complaint	1 (<1)	0 (0)	1 (<1)	
Documentation	15 (7)	5 (11)	10 (6)	
Fall	7 (3)	1 (2)	6 (4)	
Health care associated infection/infestation	1 (<1)	0 (0)	1 (<1)	
Medical device/equipment/property	12 (6)	2 (5)	10 (6)	
Medication/intravenous fluid	86 (41)	21 (48)	65 (39)	
Nutrition	0 (0)	0 (0)	0 (0)	
Organization management/service	3 (1)	1 (2)	2 (1)	
Pathology/laboratory	5 (2)	1 (2)	4 (2)	
Pressure ulcer	12 (6)	3 (7)	9 (5)	

Data are *n* (%) unless otherwise stated.

## DISCUSSION

To our knowledge, this is the first study examining the quality and safety outcomes for inpatient children with intellectual disability admitted to a tertiary paediatric health care organization using routinely collected inpatient data. We used the electronic medical record to undertake a retrospective chart review of 1021 randomly selected patients admitted for longer than 23 hours to two tertiary children’s hospitals in Sydney, Australia in 2017. Our analysis found the prevalence of children with intellectual disability or developmental delay in the admitted population was just under 14%; this represents 1 in 7 admissions of longer than 23 hours. With the prevalence of children with intellectual disability in the general Australian population estimated at 4.5%,^2^ our findings demonstrate that Australian children with intellectual disability have a high health care utilization rate and therefore have more quality and safety experiences than their peers.

Our finding of higher health care utilization in the intellectual disability population compared to the general population is not unexpected and is consistent with health care utilization studies undertaken elsewhere. Glover et al. found English children with intellectual disabilities had longer hospital stays and more episodes of care than children without intellectual disability.^7^ Similar findings regarding high health care utilization in both children and adults with intellectual disability have been reported in the Australian context.^6^, ^15^ There is evidence that children and adults with intellectual disability are more likely than those without intellectual disability to be hospitalized for conditions that can be managed in primary care settings, such as asthma and diabetes.^26^

Our previous work demonstrated children with intellectual disability are susceptible to poor care quality and patient safety experiences when in hospital.^13^ In this study we have used a variety of quality and safety metrics and patient demographics to explore quality and safety: length of stay, cost of admission, and reported incidents. We again found that children with intellectual disability had a median length of stay that was almost a day longer than children without intellectual disability. We used length of stay as a quality indicator as it has been found to be associated with reported adverse events in paediatric settings^23^ and is an indicator of care quality and hospital efficiency.^22^ Prolonged length of stay for children with intellectual disability has been reported by others^26^, ^27^, ^28^, ^29^ and may be assumed to be attributed to comorbid and complex health conditions. However, there is also mounting evidence that children and young people with intellectual disability also have higher mortality rates than their peers, and much of this difference is amenable to enhanced care quality.^14^, ^15^, ^16^

One of the reasons for the disparity in length of stay that we have demonstrated is that children with intellectual disability may need a longer hospital stay because of existing comorbid conditions.^4^, ^5^ This requires further investigation that is beyond the scope of this paper. Given this increased length of stay for children with intellectual disability it is essential that we optimize the quality of care experiences for them through partnerships with parents and a shared understanding of their children’s needs.^12^

To our knowledge, this study is the first to report and describe rates of reported clinical incidents specifically in admitted children with intellectual disability. We found the rate of reported clinical incidents for all admissions to be 10.4% of admissions, which is consistent with the evidence base over the past 20 years.^8^ Although in the paediatric population the incidence of adverse events is variable, rates of 9.2%^9^ to 36.7%^30^ of admissions have been reported. While in our study children with intellectual disability had more admissions with at least one reported clinical incident compared to children without intellectual disability, this population also had a longer median length of stay. Targeted research to explore the type, context, and time of clinical incidents for children with intellectual disability would be beneficial to understand if frequency and length of admissions are contributing factors.

In total, 48% of all reported clinical incidents for children with intellectual disability in our study were related to medication/intravenous fluid. The Australian CareTrack study, involving the medical records of 6689 children in three Australian states, found 48% of medication/intravenous fluid related incidents in paediatric patients across primary, secondary, and tertiary care settings, using a global trigger tool.^31^ Others have found children are particularly susceptible to medication‐related errors when admitted to hospital because of the variable dosing required to account for weight and growth differences,^32^, ^33^, ^34^ which suggests children with complex or chronic medical conditions requiring regular medication may have increased risk of medication errors when in hospital. When considered alongside the CareTrack study, our findings contribute toward the enhanced understanding of risk factors related to medication incidents in paediatric health care and provide direction for targeted research and service improvements involving hospitalized children and young people with intellectual disability. Adaptions to existing paediatric care coordination models, such as complex care navigators^35^ and specialist nursing teams,^36^ and the consistent use of supportive resources such as hospital passports^37^ are some examples which may enhance the quality and safety experience for inpatient children and young people with intellectual disability.

Partnering with patients, parents, and families may increase the rate of adverse event detection,^38^ and hence enhance learning and improvement opportunities. Including parents/carers in incident reporting processes are particularly crucial to reduce medication‐related errors, as parents typically manage their child’s daily medication needs outside of hospital.^23^ In addition, for people with intellectual disability in hospital, medication reconciliation processes are of particular importance from admission through to discharge.^27^ Considering the findings from our study, medication reconciliation and shared care models may be especially relevant for children with intellectual disability, and consequently medication‐related clinical incidents in inpatient paediatric settings. Further exploration of the role of parental presence in protecting children with intellectual disability from inpatient harms due to clinical incidents may lead to new learnings and enhanced health care quality and safety.

Our findings regarding rates of clinical incidents showed children with intellectual disability are not at increased risk of reported clinical incidents when in hospital. However, it is also possible that our reported incident findings may be an underestimate of the true number of clinical incidents in children with intellectual disability. In a study of parent‐reported patient safety incidents in inpatient children, Khan et al. found that of the 37 safety incidents reported by 34 parents and subsequentially validated by medical reviewers, 43% (*n*=10) were not documented in the medical record.^23^ Our previous reviews have shown that health care staff rely on parental presence when caring for a child with intellectual disability, leaving parents to attend to the care needs of their child with intellectual disability.^12^, ^13^ Combined with the findings from this current study, it is possible that parents of children with intellectual disability may be intervening to prevent harm to their child, and these actions may be unseen by clinical staff. This has been noted in a previous study of risk management by parents of their children with severe learning disabilities which found that parents hold a boundless sense of responsibility for their child; while some may oscillate between protecting their child from harm and allowing some autonomy for their child, they were mostly unwilling to risk their child’s safety in settings where they were unfamiliar, such as in hospital.^39^ This suggests parental presence may be protective against poor quality and safety experiences for children with intellectual disability in hospital.

While length of stay, cost of admission, and clinical incidents reflect impact and costs to the organization and broader health system, what is not captured here are the psychosocial, economic, and lost educational costs of hospital stays for children with intellectual disability and their families. For these children and their families, having a prolonged length of stay may have much greater clinical and social significance than can be detected through statistical analysis. In the present study, children with intellectual disability were more likely to live in a rural location than their peers, and one quarter of children with intellectual disability had more than one admission to hospital during the study period. In 2016, Mumford et al. undertook a survey of families admitted to the same health care organization where we conducted our study to determine the financial and productivity costs for families when a child is hospitalized. Their study found each patient day had a mean non‐medical cost for a parent of A$125 and required the parent to take on average 1.1 days off work, with greater costs incurred for those living in rural and remote locations.^40^ However, as the authors note, their findings are likely an underestimate as they do not reflect the cost of caring for a child at home after discharge, or the long‐term implications for employment for parents of children with chronic illness. Researchers in Victoria, Australia, in a study conducted between 2013 and 2016, explored the societal cost of childhood intellectual disability, calculating the costs incurred by caregivers of children with intellectual disability as A$382 per month.^41^ The importance of improving the health care quality and safety experiences of children with intellectual disability and their families becomes even more critical in the context of these findings.

### Limitations

Retrospective chart reviews using routinely collected hospital and health care data have been used by others to identify patient cohorts and assess patient factors associated with increased risk of adverse events in hospitalized patients, though the reliability of these studies is limited by the quality of documentation.^42^ However, extracting patient data from medical records is superior to alternatives such as incident reports alone.^43^ Furthermore, although our study utilized electronic medical records which enabled rapid and complete patient data extraction, determining if a child had intellectual disability was resource intensive, necessitating manual searches of each record. In addition, a recent Australian study found hospital mortality data is unreliable for identifying all known children with intellectual disability.^44^

Geographical barriers to tertiary health care may impact on the time spent in hospital. In our study children with intellectual disability were more likely to be from rural locations than children without intellectual disability; this may have contributed to the difference in length of stay between the two groups overall. The small number of children from rural areas meant that meaningful analysis comparing length of stay for children with and without intellectual disability from rural areas was not possible. Further research focussed on children with and without intellectual disability from rural areas may tease out any specific quality and safety considerations for these children and their families.

Compounding the challenge of reliable identification is the variety of terms that may be used to document intellectual disability in the medical record. Poor coding of intellectual disability and non‐disclosure of intellectual disability are well described barriers to reliable identification of this population in health care data.^45^, ^46^ Furthermore, formal evaluation for intellectual disability or developmental delay on admission is not routine in this setting; it may be conducted if a delay is identified or suspected on presentation or if developmental assessment is a part of the reason for admission. In general, most admitted children would have a growth and development screen and be referred for further assessment if indicated. Therefore, while our method was time consuming and the prevalence rate of 13.9% is likely an underestimate, the results demonstrate the importance of consistent definitions and differentiating children with intellectual disability or developmental delay from children with chronic illness or complex care needs.^47^

Within our data set we identified seven patients who had their admission type changed during the course of an admission, but not discharged. This resulted in these patients being documented as having two separate but sequential admissions without leaving hospital. We have no way to tell if these admissions are genuinely separate admissions or an administrative change so we did not make any alterations to the data set. Finally, because of the subjective nature of clinical incident reporting and because some admissions had more than one incident reported, we could not reliably analyse the incident data according to the number of incidents. While children with intellectual disability were overrepresented in the clinical incident data, we lacked the admission level detail to undertake further statistical analysis.

### Future directions

Our findings have exposed a significant gap in the understanding of how and why quality and safety outcomes differ in children with and without intellectual disability. Of particular importance is the need for parent and patient perspective on clinical incidents. Developing accessible methods to enable children with intellectual disability and their parent/carers to contribute directly to reporting their quality and safety experience of hospital will enhance understanding of the safety risks for this group of children.

A significant challenge is that intellectual disability is not, in itself, a medical condition though many conditions associated with intellectual disability require treatment from health professionals. Consistent research has found poor health outcomes and premature mortality in people with intellectual disability,^14^, ^15^ which is also evident in childhood.^16^ However, children with intellectual disability are routinely included in cohorts with children with complex care needs, special care needs, or chronic illness.^48^ Clegg and Bigby argue that this dedifferentiation, or including of people with intellectual disability in broader disability or chronic health populations, enhances disadvantages for this group through impaired representation and marginalization, and reduced educational and health care specialization and social care.^47^ Our study indicates that further exploration of health care experiences for these children as a unique group would greatly enhance our understanding of health care quality and safety deficiencies, for the benefit of all children going to hospital.

The findings from this current study indicate further investigation into the patient safety risks and strategies to enhance care quality experiences for children with intellectual disability are well overdue. The absence of a reliable method for identifying admissions of children with intellectual disability is a key challenge for understanding the full extent of the experience. ICD‐10 codes are a helpful guide for identifying children with specific syndromes or conditions associated with intellectual disability but in this context they are used for funding purposes. Reliable identification of intellectual disability would facilitate further research to investigate if there are disparities in quality and safety outcomes between diagnoses in children with and without intellectual disability.

Electronic medical record systems in New South Wales lack reliable methods to identify or flag patients with intellectual disability when they access health care services. However, flags or alerts are not a panacea for poor quality care; in a review of intellectual disability electronic medical record alerts in the English National Health Service, Kenten et al. found that flags and alerts are only helpful if they trigger a response or action for staff to change and adapt care delivery or practice.^46^

Whatever the method used, identification of this group of children when they are admitted to hospital can only be effective if it results in direct action by the organization and/or clinical staff to partner with the child and their parent/carer to make necessary adaptions to care delivery. Furthermore, health care organizations can consider ways to meaningfully and actively include children with intellectual disability and their parents/carers in the design and development of interventions to enhance health care delivery. Listening to and acting on the voice of children with intellectual disability and their parents/carers is a key step to understanding and improving the experience of hospital for these children.

## CONCLUSION

This research has highlighted that children who have intellectual disability experience inequities in quality and safety. We have identified that there is further investigation required to understand the safety risks for children with intellectual disability. This group are an important cohort for health systems to identify, report on, and find methods to capture and include their quality and safety experiences of health care to inform targeted improvements to health care delivery for all children in hospital.

## CONFLICT OF INTEREST

The authors have stated that they had no interests which might be perceived as posing a conflict or bias.

## Supporting information


**Figure S1:** Flowchart for determining prevalence of children with intellectual disability in admissions in 2017

**Appendix S1:** Notes on randomization and allocation of children to the intellectual disability or developmental delay group

## Data Availability

The data that support the findings of this study are available on request from the corresponding author. The data are not publicly available due to privacy or ethical restrictions.

## References

[1] Maulik PK , Mascarenhas MN , Mathers CD , Dua T , Saxena S . Prevalence of intellectual disability: a meta‐analysis of population‐based studies. Res Dev Disabil 2011; 32: 419–36. 21236634 10.1016/j.ridd.2010.12.018

[2] Australian Bureau of Statistics . Disability, ageing and carers, Australia: summary of findings [Internet]. Canberra: Australian Government; 2018. https://www.abs.gov.au/ausstats/abs@.nsf/Latestproducts/4430.0Main%20Features152018?opendocument&tabname=Summary&prodno=4430.0&issue=2018&num=&view. Accessed 25 Jul 2020.

[3] World Health Organization . Definition: intellectual disability [Internet]. Geneva: WHO. https://www.euro.who.int/en/health‐topics/noncommunicable‐diseases/mental‐health/news/news/2010/15/childrens‐right‐to‐family‐life/definition‐intellectual‐disability. Accessed 25 Jul 2020.

[4] Oeseburg B , Dijkstra GJ , Groothoff JW , Reijneveld SA , Jansen DEMC . Prevalence of chronic health conditions in children with intellectual disability: a systematic literature review. Intellect Dev Disabil 2011; 49: 59–85. 21446871 10.1352/1934-9556-49.2.59

[5] Einfeld SL , Ellis LA , Emerson E . Comorbidity of intellectual disability and mental disorder in children and adolescents: a systematic review. J Intellect Dev Disabil 2011; 36: 137–43. 21609299 10.1080/13668250.2011.572548

[6] Bebbington A , Glasson E , Bourke J , De Klerk N , Leonard H . Hospitalisation rates for children with intellectual disability or autism born in Western Australia 1983–1999: a population‐based cohort study. BMJ Open 2013; 3: e002356. 10.1136/bmjopen-2012-002356 PMC3586131 23449747

[7] Glover G , Williams R , Tompkins G , Oyinlola J . An observational study of the use of acute hospital care by people with intellectual disabilities in England. J Intellect Disabil Res 2019; 63: 85–99. 30221429 10.1111/jir.12544

[8] National Patient Safety Foundation . Free from harm: accelerating patient safety improvement fifteen years after *To Err is Human* . Boston, MA: National Patient Safety Foundation; 2015.

[9] Matlow AG , Baker G , Flintoft V , Cochrane D , Coffey M , Cohen E , et al. Adverse events among children in Canadian hospitals: the Canadian Paediatric Adverse Events Study. CMAJ 2012; 184: E709–18. 22847964 10.1503/cmaj.112153 PMC3447037

[10] Stockwell DC , Landrigan CP , Toomey SL , Loren SS , Jang J , Quinn JA , et al. Adverse events in hospitalized pediatric patients. Pediatrics 2018; 142: 1. 10.1542/peds.2017-3360 PMC6317760 30006445

[11] Stockwell DC , Landrigan CP , Toomey SL , Westfall MY , Liu S , Parry G , et al. Racial, ethnic, and socioeconomic disparities in patient safety events for hospitalized children. Hosp Pediatr 2019; 9: 1–5. 30509900 10.1542/hpeds.2018-0131 PMC6600809

[12] Mimmo L , Woolfenden S , Travaglia J , Harrison R . Partnerships for safe care: a meta‐narrative of the experience for the parent of a child with intellectual disability in hospital. Health Expect 2019; 22: 1199–212. 31560839 10.1111/hex.12968 PMC6882263

[13] Mimmo L , Harrison R , Hinchcliff R . Patient safety vulnerabilities for children with intellectual disability in hospital: a systematic review and narrative synthesis. BMJ Paediatr Open 2018; 2: e000201. 10.1136/bmjpo-2017-000201 PMC5843001 29637187

[14] Heslop P , Blair PS , Fleming P , Hoghton M , Marriott A , Russ L . The Confidential Inquiry into premature deaths of people with intellectual disabilities in the UK: a population‐based study. Lancet 2014; 383: 889–95. 24332307 10.1016/S0140-6736(13)62026-7

[15] Reppermund S , Heintze T , Srasuebkul P , Reeve R , Dean K , Smith M , et al. Health and wellbeing of people with intellectual disability in New South Wales, Australia: a data linkage cohort. BMJ Open 2019; 9: e031624. 10.1136/bmjopen-2019-031624 PMC6773320 31575581

[16] Smith GS , Fleming M , Kinnear D , Henderson A , Pell JP , Melville C , et al. Rates and causes of mortality among children and young people with and without intellectual disabilities in Scotland: a record linkage cohort study of 796 190 school children. BMJ Open 2020; 10: e034077. 10.1136/bmjopen-2019-034077 PMC7418667 32773385

[17] World Health Organization . ICD‐10: international statistical classification of diseases and health related problems: tenth revision [Internet]. Geneva: WHO; 2004. http://www.who.int/iris/handle/10665/42980. Accessed 15 May 2018.

[18] Mimmo L , Woolfenden S , Travaglia J , Harrison R . Creating equitable healthcare quality and safety for children with intellectual disability in hospital. Child Care Health Dev 2020; 46: 644–9. 32468634 10.1111/cch.12787 PMC7496444

[19] Tuffrey‐Wijne I , Goulding L , Gordon V , Abraham E , Giatras N , Edwards C , et al. The challenges in monitoring and preventing patient safety incidents for people with intellectual disabilities in NHS acute hospitals: evidence from a mixed‐methods study. BMC Health Serv Res 2014; 14: 432. 25253430 10.1186/1472-6963-14-432 PMC4263117

[20] Mithyantha R , Kneen R , McCann E , Gladstone M . Current evidence‐based recommendations on investigating children with global developmental delay. Arch Dis Child 2017; 102: 1071–6. 29054862 10.1136/archdischild-2016-311271 PMC5738593

[21] Matson JL , Shoemaker M . Intellectual disability and its relationship to autism spectrum disorders. Res Dev Disabil 2009; 30: 1107–14. 19604668 10.1016/j.ridd.2009.06.003

[22] Organisation for Economic Cooperation and Development . Health at a glance 2019: OECD indicators. Paris: OECD; 2019.

[23] Khan A , Furtak SL , Melvin P , Rogers JE , Schuster MA , Landrigan CP . Parent‐reported errors and adverse events in hospitalized children. JAMA Pediatr 2016; 170: e154608. 26928413 10.1001/jamapediatrics.2015.4608 PMC5336322

[24] Slawomirski L , Auraaen A , Klazinga NS . The economics of patient safety. Paris: OECD; 2017.

[25] Clinical Excellence Commission . Incident management policy. North Sydney: NSW Health; 2020.

[26] Dunn K , Hughes‐McCormack L , Cooper S‐A . Hospital admissions for physical health conditions for people with intellectual disabilities: systematic review. J Appl Res Intellect Disabil 2018; 31: 1–10. 10.1111/jar.12360 28467010

[27] Iacono T , Bigby C , Unsworth C , Douglas J , Fitzpatrick P . A systematic review of hospital experiences of people with intellectual disability. BMC Health Serv Res 2014; 14: 505. 25344333 10.1186/s12913-014-0505-5 PMC4210514

[28] Baker AB , Farhood Z , Brandstetter KA , Teufel RJ , LaRosa A , White DR . Tonsillectomy in children with Down syndrome: a national cohort of inpatients. Otolaryngoly Head Neck Surg 2017; 157: 499–503. 10.1177/0194599817711377 28762292

[29] Williams K , Leonard H , Tursan d’Espaignet E , Colvin L , Slack‐Smith L , Stanley F . Hospitalisations from birth to 5 years in a population cohort of Western Australian children with intellectual disability. Arch Dis Child 2005; 90: 1243–8. 16301550 10.1136/adc.2004.062422 PMC1720232

[30] Kirkendall E , Kloppenborg E , Papp J , White D , Frese C , Hacker D , et al. Measuring adverse events and levels of harm in pediatric inpatients with the global trigger tool. Pediatrics 2012; 130: e1206. 23045558 10.1542/peds.2012-0179

[31] Hibbert PD , Runciman WB , Carson‐Stevens A , Lachman P , Wheaton G , Hallahan AR , et al. Characterising the types of paediatric adverse events detected by the global trigger tool – CareTrack Kids. J Patient Saf Risk Manag 2020; 25: 239–49.

[32] Grissinger M . Medication errors affecting pediatric patients: unique challenges for this special population. Patient Saf Advis 2015; 12: 96–102.

[33] Taitz J . Building a culture of safety in paediatric and child health. Curr Treat Options Pediatr 2015; 1: 9.

[34] Meurer JR , Yang H , Guse CE , Scanlon MC , Layde PM . Medical injuries among hospitalized children. Qual Saf Health Care 2006; 15: 202–7. 16751471 10.1136/qshc.2005.015412 PMC2464854

[35] Zurynski Y , Altman L , Breen C , Woolfenden S . Care coordination for children with chronic and complex conditions in Australia: significant benefits for patients and their families. Int J Integr Care 2018; 18: 113.

[36] Castles A , Bailey C , Gates B , Sooben R . Experiences of the implementation of a learning disability nursing liaison service within an acute hospital setting: a service evaluation. Br J Learn Disabil 2014; 42: 272–81.

[37] Northway R , Rees S , Davies M , Williams S . Hospital passports, patient safety and person‐centred care: a review of documents currently used for people with intellectual disabilities in the UK. J Clin Nurs 2017; 26: 5160–8. 28881074 10.1111/jocn.14065

[38] Khan A , Coffey M , Litterer KP , Baird JD , Furtak SL , Garcia BM , et al. Families as partners in hospital error and adverse event surveillance. JAMA Pediatr 2017; 171: 372–81. 28241211 10.1001/jamapediatrics.2016.4812 PMC5526631

[39] Oulton K , Heyman B . Devoted protection: How parents of children with severe learning disabilities manage risks. Health Risk Soc 2009; 11: 303–19.

[40] Mumford V , Baysari MT , Kalinin D , Raban MZ , McCullagh C , Karnon J , et al. Measuring the financial and productivity burden of paediatric hospitalisation on the wider family network. J Paediatr Child Health 2018; 54: 987–96. 29671913 10.1111/jpc.13923 PMC6635734

[41] Arora S , Goodall S , Viney R , Einfeld S . Societal cost of childhood intellectual disability in Australia. J Intellect Disabil Res 2020; 64: 524–37. 32329168 10.1111/jir.12732

[42] Aranaz‐Andres JM , Limon R , Mira JJ , Aibar C , Gea MT , Agra Y . What makes hospitalized patients more vulnerable and increases their risk of experiencing an adverse event? Int J Qual Health Care 2011; 23: 705–12. 21896634 10.1093/intqhc/mzr059

[43] Sari AB‐A , Sheldon TA , Cracknell A , Turnbull A , Dobson Y , Grant C , et al. Extent, nature and consequences of adverse events: results of a retrospective casenote review in a large NHS hospital. Qual Saf Health Care 2007; 16: 434–9. 18055887 10.1136/qshc.2006.021154 PMC2653177

[44] Bourke J , Wong K , Leonard H . Validation of intellectual disability coding through hospital morbidity records using an intellectual disability population‐based database in Western Australia. BMJ Open 2018; 8: e019113. 10.1136/bmjopen-2017-019113 PMC5786126 29362262

[45] Emerson E , Hatton C . Health inequalities and people with intellectual disabilities. Cambridge, UK: Cambridge University Press; 2014.

[46] Kenten C , Wray J , Gibson F , Russell J , Tuffrey‐Wijne I , Oulton K . To flag or not to flag: identification of children and young people with learning disabilities in English hospitals. J Appl Res Intellect Disabil 2019; 32: 1176–83. 31095841 10.1111/jar.12608 PMC6852602

[47] Clegg J , Bigby C . Debates about dedifferentiation: twenty‐first century thinking about people with intellectual disabilities as distinct members of the disability group12. Res Pract Intellect Dev Disabil 2017; 4: 80–97.

[48] Graham RJ , Wachendorf MT , Burns JP , Mancuso TJ . Successful and safe delivery of anesthesia and perioperative care for children with complex special health care needs. J Clin Anesth 2009; 21: 165–72. 19464608 10.1016/j.jclinane.2008.06.033

