# Supplementary material for: Inequities in quality and safety outcomes for hospitalized children with intellectual disability
Source: Dev Med Child Neurol. 2021 Sep 25;64(3):314–22. doi: 10.1111/dmcn.15066 (PMC9293445; doi:10.1111/dmcn.15066)
Supplement: Supplementary file 1 — Figure S1: Flowchart for determining prevalence of children with intellectual disability in admissions in 2017 [file DMCN-64-314-s002.docx]

21,337 admissions greater than 23 hours in 2017

Unique patient medical record numbers (MRN) extracted into dataset

MRN from every 16^th^ row selected for inclusion

**No**

**Yes**

893 patients

(87.7%)

125 patients

(12.3%)

1018 patients

1021 randomly selected patients

**No**

**Yes**

**Intellectual disability/**

**developmental delay?**

**Intellectual disability/**

**developmental delay?**

190 admissions (13.9%)

1177 admissions (86.1%)

1367 admissions

3 excluded as not admitted to ward

*Figure S1: Flowchart for determining prevalence of children with intellectual disability in admissions in 2017.*
