# Supplementary material for: Inequities in quality and safety outcomes for hospitalized children with intellectual disability
Source: Dev Med Child Neurol. 2021 Sep 25;64(3):314–22. doi: 10.1111/dmcn.15066 (PMC9293445; doi:10.1111/dmcn.15066)
Supplement: Supplementary file 2 — Appendix S1: Notes on randomization and allocation of children to the intellectual disability or developmental delay group [file DMCN-64-314-s001.docx]

**APPENDIX ONE**

Notes on randomisation and allocation of children to the intellectual disability or developmental delay group.

Randomisation

The randomisation was done by the medical record department and based on individual hospital medical record numbers (MRN) not by admission. The method used by the data analyst in the medical record department was:

1. Extract the MRNs for all patients admitted for greater than 23 hours from 01/01/17 to 31/12/17;
2. Select every 16^th^ MRN row in the dataset;
3. Extract the details for each admission in 2017 associated with each individual MRN.

Allocation of children to the intellectual disability or developmental delay group

Children under 6 years or not yet in school were only allocated to the developmental delay group if:

1. The child had a condition associated with intellectual disability (for example down syndrome or fragile X syndrome);
2. There was documentation that the child had significant/global developmental delay – usually documented following a developmental assessment with a validated tool.

Determining which children went into the intellectual disability group was decided on documentation in the medical record not just individual assessment. All children were assumed to not have intellectual disability unless there was clear documented evidence to say otherwise. In the absence of sufficient documented evidence of intellectual disability or global developmental delay a child would be allocated to the no intellectual disability cohort. Therefore, only children with either documented global developmental delay or documented intellectual disability were allocated to the intellectual disability cohort.

The developmental evaluations were used as a guide to identifying potential children with global developmental delay or intellectual disability; the medical record reviewer, LM, did not use the assessments to make a diagnosis, rather the record reviewer looked through the assessments to find documented evidence that the child did or did not have global developmental delay or intellectual disability.

Determining intellectual disability in younger children from a chart review is challenging. As described above, we sought specific documentation of intellectual disability before allocating a child to this cohort. In the case of children under 1yr, or did not have any developmental assessments recorded, this was based on documented evidence in the clinical notes that the child had a condition that includes intellectual disability such as Down Syndrome, Angelman Syndrome, Fragile X Syndrome. There were some patients whose medical record notes suggested global developmental delay however if there was no specific documentation that this was the case the child was not put in the intellectual disability cohort.
